# Short interpregnancy interval following reproductive coercion: A prospective cohort study of pregnant Ethiopian women

**DOI:** 10.1002/pmf2.70079

**Published:** 2025-07-31

**Authors:** Jessica L. Dozier, Shannon N. Wood, Nancy Perrin, Solomon Shiferaw, Robel Yirgu, Linnea A. Zimmerman

**Affiliations:** ^1^ Department of Population Family and Reproductive Health Johns Hopkins Bloomberg School of Public Health Baltimore Maryland USA; ^2^ Department of Systems Populations and Leadership School of Nursing University of Michigan Ann Arbor Michigan USA; ^3^ School of Nursing Johns Hopkins University Baltimore Maryland USA; ^4^ School of Public Health Addis Ababa University Addis Ababa Ethiopia

**Keywords:** intimate partner violence, maternal health, pregnancy coercion, pregnancy spacing, reproductive autonomy, reproductive coercion, short interpregnancy interval

## Abstract

**Background:**

Short interpregnancy intervals (IPIs) (< 12 months) remain a public health concern in low‐ and middle‐income countries due to their association with adverse maternal and neonatal outcomes. Reproductive coercion (RC), which involves intimate partner behaviors that interfere with women's reproductive decisions, undermines autonomy, and increases the risk of unintended pregnancy. This exploratory study examines the relationship between RC and short IPIs.

**Methods:**

Data come from a prospective cohort (2019–2021) of pregnant Ethiopian women aged 15–49, followed up to 1 year postpartum (*N* = 1935). Kaplan–Meier estimators and survival‐time models assessed relationships between RC—experienced before the index pregnancy, measured dichotomously (yes/no) and by severity (none, less severe, more severe)—and having an IPI < 12 months following a live birth.

**Findings:**

Overall, 27.3% of women experienced RC before their index pregnancy, and 4.4% had a subsequent pregnancy within 12 months of giving birth (IPI range: 3–12 months). Among women who experienced any RC, less severe RC, or more severe RC, 5.8%, 5.5%, and 6.2%, respectively, had short IPIs, compared to 3.8% of those who did not experience RC. Any RC was associated with a marginally higher risk of short IPI compared to no RC (HR: 1.55, 95% CI: 0.97–2.48, *p* = 0.07). Disaggregating by RC severity, there was a small, non‐significant increase in risk among women who experienced less severe RC (HR: 1.44, 95% CI: 0.80–2.59; *p* = 0.23), and a marginally higher risk of short IPI among women who experienced more severe RC behaviors (HR: 1.72, 95% CI 0.97–3.04, *p* = 0.06) compared to women who did not experience RC.

**Interpretation:**

Women who experience more severe RC may face an elevated risk of short IPI. The observed dose‐response trend between RC severity and short IPI warrants further investigation to better understand how RC may impact pregnancy spacing. Clarifying the role of RC in short IPIs could help identify factors that limit women's reproductive autonomy.

## 1 INTRODUCTION

Short interpregnancy intervals (IPIs), defined as pregnancies occurring within 24 months of a previous birth, remain a significant global health challenge, particularly in low‐ and middle‐income countries, where up to one‐third of all subsequent births occur within this timeframe [1, 2]. In sub‐Saharan Africa, approximately one in five non‐first births occur within 24 months of a prior birth [1] which contributes to a burden of maternal and child morbidity and mortality that exceeds that of many other regions [3, 4, 5, 6]. Closely spaced pregnancies are associated with an increased risk of poor maternal outcomes, including uterine rupture, premature rupture of membranes, and maternal mortality [7, 8], as well as neonatal complications such as preterm birth, low birth weight, and neonatal death [8, 9, 10, 11]. Very short IPIs (less than 12 months) pose even greater risks. These pregnancies are associated with a greater risk of perinatal death compared to those spaced 12–24 months apart [12]. A meta‐analysis of 113 Demographic and Health Surveys from 46 countries found that pregnancies conceived less than 6 months after a previous birth are more than twice as likely to end in perinatal death compared to those with an IPI of 18–23 months [13]. Pregnancies conceived 6–11 months after a birth also had nearly double the risk [13]. To promote safer maternal and neonatal outcomes, the World Health Organization recommends delaying pregnancy for at least 24 months following a live birth [14].

The initiation of contraception within the first year after childbirth, called postpartum family planning (PPFP), is a critical strategy for achieving healthy birth spacing and reducing the risks associated with short IPIs [15]. Despite its importance, many individuals face barriers to accessing and using effective contraception during the postpartum period, including limited availability, restrictive sociocultural norms, and partner opposition [16, 17, 18, 19, 20]. In sub‐Saharan Africa, where unmet need for postpartum contraception remains high [21], strengthening access to PPFP is essential to improving maternal and neonatal health outcomes. In many pronatalist settings across the region, patriarchal norms and gender‐inequitable power dynamics enable men to exert control over decisions related to contraceptive use, pregnancy timing, and family size [16, 17, 18, 19, 20]. Against this backdrop, unmet need for contraception is as high as 88% in some countries [21].

Moreover, women in sub‐Saharan Africa often prefer longer birth intervals than their partners [22], yet often experience shorter IPIs than desired [23]. Studies suggest that short IPIs are more common when male partners oppose pregnancy spacing [24] or make unilateral decisions about when to have the next child [25]. In some cases, women may fear conflict or relationship instability if they delay pregnancy longer than their partner's expectations [18]. Experiences of physical or sexual intimate partner violence may further constrain women's ability to achieve their desired pregnancy spacing and limit their reproductive autonomy [26, 27].

Partner‐perpetrated reproductive coercion (RC) is one way women's reproductive autonomy may be undermined. RC refers to behaviors by an intimate partner aimed at controlling decisions about contraception and pregnancy [28]. Although it has been largely overlooked as a potential determinant of short IPI, RC includes tactics such as sabotaging contraception, interfering with access to family planning services, pressuring pregnancy against a woman's wishes, and attempting to control pregnancy outcomes [28, 29, 30]. Studies link RC to decreased likelihood of contraceptive use [31, 32] and increased risk of unintended pregnancy [32, 33]. Qualitative research from sub‐Saharan Africa suggests that men may coerce pregnancy in part to uphold social status associated with having children [34] and that RC may continue until a woman has the number of children *her partner* desires [35, 36]. In Kenya, interviews with family planning clients reveal that some women experience partner interference in postpartum contraception and threats of infidelity if they delay pregnancy after childbirth [35]. Despite growing evidence on RC, its relationship to pregnancy spacing remains unexamined. Understanding this interplay is particularly important in settings characterized by gender inequity and high fertility, where targeted interventions are needed to support women's autonomy and reproductive self‐determination.

Ethiopia offers a critical context in which to explore this relationship. Many women experience closely spaced pregnancies, and inequitable gender norms can constrain women's reproductive decision‐making. Half of all pregnancies following a live birth occur within 24 months [2] and a substantial proportion occur even sooner. For example, in the Amhara region, 10% of women in a facility‐based sample [37] and 5% in a community‐based sample had IPIs of less than 12 months [38]. Moreover, one in five partnered women aged 15–49 report experiencing RC in the past year [31], which may undermine women's ability to plan and space their pregnancies and contribute to adverse health outcomes.

Given the limited evidence on how RC may influence pregnancy spacing, the current exploratory study examines the relationship between RC and short IPI in a prospective cohort of Ethiopian women followed from pregnancy through the first year postpartum. Specifically, we assess relationships between RC and (1) the occurrence of a repeat pregnancy within 12 months of a live birth and (2) the time to a repeat pregnancy.

## 2 METHODS

### 2.1 Study setting

Despite substantial progress over the past two decades, Ethiopia still has one of the highest neonatal (26 deaths per 1000 live births) and infant (43 deaths per 1000 live births) mortality rates in the world [39]. Short IPIs contribute to this burden, as half of all pregnancies after a live birth occur before the recommended 24‐month spacing interval [2]. Infants born following a short IPI of less than 24 months are twice as likely to die compared to those born after longer intervals [4].

An estimated four in five women 0–24 months postpartum have an unmet need for family planning [2]. At one year postpartum, most Ethiopian women (71%) want to wait at least two years before having more children, though more than half (57%) are not using contraception [40]. Partner opposition is frequently cited as a contributor to nonuse of PPFP [41, 42, 43] and Ethiopian women often have shorter birth intervals than they prefer [44]. Notably, more married men (22%) than women (18%) aged 15–49 express a desire to have another child within two years of giving birth [44].

### 2.2 Data source and study design

Data come from the Performance Monitoring for Action‐Ethiopia (PMA‐Ethiopia) 2019–2021 cohort, which includes a regionally representative sample of pregnant and recently postpartum women ages 15–49 from Addis Ababa, Afar, Amhara, Oromia, SNNP (Southern Nations, Nationalities and Peoples), and Tigray [45].^a^ PMA‐Ethiopia employed a multistage stratified survey design, sampling 217 clusters or enumeration areas (EAs) from the six regions that comprise 91% of Ethiopia's population [45]. EAs were selected with probability proportional to size, with urban/rural stratification in Amhara, Oromia, Tigray, and SNNP. Following a household census, women aged 15–49 were screened to identify women who were pregnant or less than six weeks postpartum. Eligible consenting women completed a baseline interview and were reinterviewed at approximately 6 weeks, 6 months, and 1 year postpartum. Surveys were translated into three local languages (Amharic, Afan Oromia, and Tigringya), and all data were collected between October 2019 and August 2021 by local interviewers using mobile technology. Survey implementation was managed by the Johns Hopkins Bloomberg School of Public Health.

A total of 2868 (99.6% response rate) cohort‐eligible women completed the baseline interview. Women over six weeks postpartum at baseline were no longer eligible for cohort enrollment and were excluded (*n* = 13), yielding an initial cohort of *n* = 2855 (2239 women were currently pregnant and 616 were less than six weeks postpartum at enrollment). Women who missed a follow‐up interview could complete the subsequent interview, provided they had not previously refused further follow‐up (e.g., a woman may have missed her six‐week and six‐month interviews but completed the one‐year postpartum interview). In total, 2367 women (82.9%) completed at least one follow‐up interview.

### 2.3 Measures

The outcome of interest was very short IPI—conception of a subsequent pregnancy within 12 months of the end of a woman's index pregnancy (i.e., the pregnancy for which women were eligible for enrollment into the cohort). Time to a subsequent pregnancy, or IPI length, was the time (in months) between the end of the index pregnancy and the start of the next pregnancy. We utilized a retrospective postpartum reproductive calendar that documented month‐by‐month occurrences of pregnancies, terminations, births, and contraceptive use over the 12 months following the birth of a woman's most recent child. Women provided reproductive histories from the first to the sixth month postpartum at the six‐month follow‐up interview and for months seven through twelve at one‐year postpartum interviews. If women did not complete the six‐month interview full 12‐month postpartum histories were collected at one‐year follow‐up.

Before analysis, the reproductive calendar underwent a thorough data‐cleaning process. First, we imputed pregnancies at the corresponding month for 29 women who indicated current pregnancy at follow‐up but did not have this information originally recorded in their reproductive calendar. In addition to the month‐by‐month postpartum reproductive calendar, interviewers also asked women about their current pregnancy status (“*Are you currently pregnant?*”) during both six‐month and one‐year follow‐up interviews. Because some women completed delayed follow‐up interviews due to a pause in data collection from April 2020 to July 2020 during the initial phase of the COVID‐19 pandemic (mean 10.1 weeks postpartum at 6‐week follow‐up [46] and 7.3 months at 6‐month interviews [47]), current pregnancy was imputed for the corresponding month. For instance, if a woman reported being pregnant at her delayed 6‐month follow‐up (7 months postpartum at the time), we imputed the pregnancy into her reproductive calendar at Month Seven, considering this information was not reflected in her initial reproductive calendar. Next, since Month One of women's reproductive calendar signifies the initial month after childbirth, we re‐coded women's postpartum reproductive histories in cases where their month‐by‐month history incorrectly began with pregnancy followed by birth. These calendars were recentered to start with the first month after birth; extra months were treated as missing. This adjustment reflected the chronological progression of pregnancy, childbirth, and postpartum events. Instances where this re‐coding occurred (*n* = 64) were deemed potential data entry errors, likely due to the intricacies of completing reproductive calendars in survey research. Lastly, women who reported a termination (*n* = 6) or a birth (*n* = 6) without a pregnancy in the preceding month in their 12‐month calendar were assumed to have been pregnant within the same month. This assumption was made due to the limitation of data collection, which allowed women to report only a single event (pregnancy, birth, termination, or (non) use of contraception) for each of the 12 months in the reproductive calendar.

The exposure of interest was RC in the year before the index pregnancy. During baseline interviews, women were asked to recall partner‐perpetrated behavior to promote pregnancy in the 12 months preceding the survey. Five items were adapted from a validated RC measure that had been previously tested in the US and later in sub‐Saharan Africa, including Ethiopia [31, 33, 48, 49]. Women were asked whether, in the past 12 months, their husband or partner (1) told them not to use family planning, (2) said he would leave them if they didn't get pregnant, (3) told them he would have a baby with someone else if they didn't get pregnant, (4) took away their family planning or kept them from getting to the clinic, or (5) hurt them physically because they did not agree to get pregnant. In *a priori* confirmatory factor analysis, items loaded onto a single factor (*pregnancy coercion;* eigenvalue = 1.60) with moderate internal consistency (Cronbach's alpha = 0.64).

Consistent with previous analyses [31, 50], RC was examined in two ways: as a binary variable (i.e., any past‐year RC, denoted by an affirmative response to any RC item) and as a categorical variable (i.e., no RC, less severe RC, and more severe RC). The categorical variable aimed to capture RC severity by distinguishing verbal discouragement of contraception (which may occur without coercive intent) from explicit interference or sabotage. Specifically, an affirmative response to only the “*told you not to use family planning*” item was classified as *less severe RC*, and any affirmative response to the remaining four items was classified as *more severe RC* (“*said he would leave you if you didn't get pregnant;” “told you he would have a baby with someone else if you didn't get pregnant;” “took away your family planning or kept you from going to the clinic;”* or *“hurt you physically because you did not agree to get pregnant.”*) This classification allowed us to separate behaviors theorized to indicate less direct coercion, like verbal discouragement, from those with clear intent to control reproductive decision‐making, like threats and physical violence for not complying with a partner's wishes.

Sociodemographic and reproductive variables were evaluated as potential confounders based on theoretical relationships between RC and very short IPI. Sociodemographic variables included region (Addis Ababa, Afar, Amhara, Oromia, SSNP, or Tigray), whether respondent lived in an urban or rural area, household wealth (binary variable indicating wealth distribution relative to the population: lowest 40% or highest 60%), relationship status (married or living with partner as if married), polygyny (partner has one wife or partner has more than one wife), women's age (categorical: < 25, 25–29, ≥30 years), religion (Orthodox, Muslim, or Protestant/Other), highest level of school attended (never attended, primary, or secondary or higher). PMA‐Ethiopia generated a composite household wealth quintile variable at the country level using principal components analysis based on household assets, livestock owned, roof and wall construction materials, toilet facilities, and water sources; the lower two quintiles (the bottom 40%) and the higher three quintiles (the top 60%) were combined in analysis to maximize power. Polygyny was measured using an item adapted from the Demographic and Health Surveys (DHS) that assessed whether a woman's partner had concurrent partners [51], “*Does your husband/partner have other wives, or does he live with other women as if married*?”

Reproductive characteristics were measured at baseline (i.e., during index pregnancy) and included parity (binary variable indicating whether the index pregnancy was a woman's first live birth: primiparous or multiparous), index pregnancy intention (binary: intended or unintended), and prospective fertility plans (binary variable measuring whether, during index pregnancy, respondent reported she wanted more children). Pregnancy intention of the index pregnancy was assessed using a question adapted from the DHS retrospective time‐based measure of pregnancy wantedness [51], (“*At the time you became pregnant, did you want to become pregnant then, did you want to wait until later, or did you not want to have any/any more children?*”). Mistimed (later) and unwanted (no more) were combined into a single unintended category to maximize statistical power. In addition, due to the past‐year timeframe of RC, gestational age (self‐reported in months) was included in models to account for women's varied exposure time to RC. Any use of a modern method of contraception (i.e., male/female sterilization, implant, intrauterine device, injectable, pills, male/female condoms, emergency contraception, and lactational amenorrhea) during the 12 months postpartum (yes/no) was assessed in descriptive analyses only and not in adjusted models due to it being on the causal pathway between RC and very short IPI.

To prevent issues of sparse data and enhance statistical power, categories of covariates with small (*n* < 20) cell sizes (i.e., wealth quintile, age, religion, parity, gestational age) were combined. For example, we combined values of the smallest and largest gestational ages (< 1% of women were one month or ten months pregnant at baseline) with the subsequent and preceding month, respectively, resulting in a range of 2–9 months.

### 2.4 Analytic sample

Analyses were restricted to women who were pregnant at baseline (*n* = 616 excluded), married or living with their partner as if married (*n* = 57 excluded), had complete RC data (*n* = 13 excluded), and completed at least one postpartum interview (*n* = 99 excluded, retention = 95.4%; attrition was non‐differential by RC exposure). Because interpregnancy intervals likely differ for women who have a live birth compared to other birth outcomes [18], women whose index pregnancy resulted in stillbirth (*n* = 39), miscarriage (*n* = 91), or abortion (*n* = 3) were further excluded. Finally, improbable reproductive calendar observations were excluded (*n* = 2; e.g., one 12‐month reproductive history included multiple pregnancies and births in the 12‐month postpartum period). The final analytic sample included *n* = 1935 women.

### 2.5 Statistical analysis

We explored weighted distributions of women's sociodemographic and reproductive characteristics using design‐based analyses that accounted for multistage cluster sampling and loss‐to‐follow‐up. Design‐based F statistics were used to compare sample characteristics and RC exposure by experience of very short IPI. To assess temporal patterns of very short IPI across the 12‐month postpartum period, we estimated the failure function (i.e., probability of a very short IPI at a specific postpartum month among women who had not already experienced a repeat pregnancy at the respective months) by constructing Kaplan–Meier failure curves. Log‐rank tests were used to compare the estimated failure rates based on RC experience. Women were censored in the analysis at the first month lost to follow‐up.

In exploratory analyses, we assessed Schoenfeld residuals to evaluate the assumption of proportional hazards for RC and predictors. Exploratory analyses revealed violations of the assumption of proportional hazards, prompting the use of a parametric regression survival‐time model with Weibull distribution to account for varying hazards over time. Crude hazard models included gestational age to account for time at risk of RC. Covariates were considered for inclusion in adjusted models based on theoretical relevance.

Exploratory analyses also examined the appropriateness of adjusting for clustering and sample weights in regression models by calculating the approximate inefficiency of applying each when making model‐based inferences [52]. Clustering was accounted for in all models, but sample weights were not applied because the use of unweighted models was determined to yield consistent parameter estimates with smaller standard errors [52]. All analyses were conducted using Stata version 16.1.

### 2.6 Ethical considerations

Trained interviewers obtained oral informed consent at study baseline and reaffirmed participants' consent to continue participation at each subsequent follow‐up interview. Data collection included screening for distress and referral to health centers for violence and reproductive health support, regardless of disclosure of violence, as is best practice for conducting research on violence against women [53]. Institutional Review Boards at Addis Ababa University and the Johns Hopkins Bloomberg School of Public Health approved study procedures.

## 3 RESULTS

### 3.1 Sample characteristics

Table 1 shows the weighted distribution of sociodemographic and reproductive characteristics of the analytic sample. Most women resided in Oromia (43.2%), SNNP (23.6%), or Amhara (20.6%), and the sample was predominantly (78.0%) from rural areas. Marriage was nearly universal (97.0%), and approximately one in ten (9.2%) women reported being in a polygynous relationship. Two‐thirds of women were between 15 and 29 years old, and the majority never attended school (42.3%) or only attended primary school (38.8%). The predominant religion was Orthodox.

**TABLE 1:** Sociodemographic and reproductive characteristics of postpartum women by short interpregnancy interval (< 12) status. Overall, 4.4% (95% CI: 3.1–6.1) of women experienced a subsequent pregnancy within 12 months of giving birth.

	Total (*N* = 1935), *n*(%)	IPI < 12 months (*N* = 82), *n* (%)	No IPI < 12 months (*N* = 1853), *n* (%)	*p* value
**Sociodemographic characteristics**				
Region				**0.40**
Addis	73 (3.8)	6 (3.1)	174 (3.8)	
Tigray	132 (6.8)	5 (1.1)	316 (7.1)	
Afar	39 (2.0)	11 (3.1)	157 (2.0)	
Amhara	399 (20.6)	18 (25.0)	315 (20.4)	
Oromia	836 (43.2)	21 (43.8)	453 (43.1)	
SNNP	456 (23.6)	21 (23.9)	438 (23.5)	
Area of Residence				0.74
Urban	426 (22.0)	29 (24.0)	694 (22.0)	
Rural	1509 (78.0)	53 (76.0)	1159 (78.0)	
Household Wealth				0.91
Lowest 40%	766 (39.6)	30 (38.8)	610 (39.6)	
Highest 60%	1169 (60.4)	52 (61.2)	1243 (60.4)	
Relationship Status				0.16
Married	1878 (97.0)	76 (93.0)	1788 (97.2)	
Living with Partner	57 (3.0)	6 (7.0)	65 (2.8)	
Polygyny				0.69
Partner has one wife	1758 (90.8)	71 (89.6)	1692 (90.9)	
Partner has more than one wife	177 (9.2)	11 (10.4)	161 (9.1)	
Age				**0**.**02**
15–24 years	667 (34.5)	25 (30.6)	618 (34.6)	
25–29 years	575 (29.7)	33 (43.5)	592 (29.1)	
≥30 years	693 (35.8)	24 (25.9)	643 (36.3)	
Religion				0.35
Orthodox	729 (37.7)	27 (28.1)	842 (38.1)	
Muslim	661 (34.2)	22 (27.6)	455 (28.2)	
Protestant/Other^a^	545 (28.2)	33 (44.3)	556 (33.7)	
Highest level of education				0.45
Never attended	819 (42.3)	26 (34.0)	729 (42.7)	
Primary	751 (38.8)	35 (45.4)	653 (38.5)	
Secondary or higher	365 (18.9)	21 (20.6)	471 (18.8)	
**Reproductive characteristics**				
Parity (inclusive of index pregnancy)				0.25
Primiparous	459 (23.7)	26 (30.1)	466 (23.4)	
Multiparous	1476 (76.3)	56 (69.9)	1387 (76.6)	
Index pregnancy intention				0.47
Intended	1243 (64.2)	62 (69.2)	1296 (64.0)	
Unintended	692 (35.8)	20 (30.6)	557 (36.0)	
Prospective fertility plans during index pregnancy				**0**.**06**
Wants no more children /don't know	574 (29.6)	18 (19.4)	516 (30.1)	
Wants more children	1361 (70.4)	64 (80.6)	1337 (69.9)	
Gestational age (in months) at enrollment, mean (SD)	5.4 (2.2)	5.2 (2.2)	5.3 (2.2)	0.45
Adopted postpartum contraception^b^				**0**.**02**
No	1089 (56.3)	61 (73.1)	986 (55.5)	
Yes	846 (43.7)	21 (26.9)	867 (44.5)	

*Note*: Bold indicates *p* < 0.05. *p* values were calculated using design‐based *F*‐statistics derived from Pearson's chi‐squared tests for categorical variables and *t*‐tests for continuous variables. Values are presented as *n* (column %). All sociodemographic and reproductive characteristics, except postpartum contraceptive use, were reported at baseline (i.e., during the index pregnancy).

Abbreviations: IPI, Interpregnancy Interval; SNNP, Southern Nations, Nationalities, and Peoples’ Region.

^a^ Other religion (*n* = 27) includes Catholic, Traditional, Wakefeta, No religion/non‐believer, and “Other.”

^b^ Includes male/female sterilization, implant, IUD, injectable, pills, male/female condoms, and emergency contraception.

Women were, on average, 5.4 months (∼23–24 weeks) pregnant at baseline and approximately a quarter (23.7%) were pregnant with their first child at index pregnancy. At baseline (i.e., during index pregnancy), more than one‐third of women reported they wanted that pregnancy later or not at all and most women (70.4%) reported they wanted more children. Less than half (43.7%) of women used any modern method of PPFP during the first year postpartum.

### 3.2 Prevalence of very short IPI by sociodemographic and reproductive characteristics

In total, 4.4% (95% Confidence Interval (CI): 3.1–6.1) of women became pregnant in the 12 months following the birth of their index pregnancy (i.e., had a very short IPI; Table 1). In bivariate analyses, very short IPI was associated with age (*p* = 0.02), prospective fertility plans (*p* = 0.06), and adoption of PPFP (*p* = 0.02). Approximately three‐quarters (74.1%) of women who had a very short IPI were under age 30, and 80.6% wanted more children at baseline. The proportion of women who adopted a modern method of PPFP was higher among women who did not have a very short IPI compared to women who did (45% vs. 27%, *p* = 0.02).

Women who experienced a very short IPI were marginally more likely to have recently given birth to their first child (i.e., primiparous at follow‐up) than women who did not experience a very short IPI (30.1% vs. 23.4%, *p* = 0.25).

### 3.3 RC and very short IPI—bivariate analyses

Overall, at the time of index pregnancy, more than one in four (27.3%) women reported experiencing any RC in the past 12 months, with 16.1% experiencing less severe RC and 11.2% experiencing more severe RC (Table 2). The proportion of very short IPI was greater among women who experienced any RC (5.8%) compared to women who had not experienced RC (3.8%; *p* = 0.09). Very short IPI increased with RC severity (6.2% among women who experienced more severe RC, 5.5% among women who experienced less severe RC, and 3.8% among those who had not experienced RC before their index pregnancy; *p* = 0.22) but was not significant. When examined by individual RC behaviors, one in seven (14.3%) women who reported their partner physically hurt them because they did not agree to get pregnant in the year before their index pregnancy had an very short IPI; in comparison, 4.2% of women who did not have this RC experience had a very short IPI (*p* = 0.04). The proportion of women experiencing very short IPI was greatest among women who experienced the most severe forms of RC; for instance, approximately one in ten (9.2%) women who reported their partner took away their family planning or kept them from going to the clinic before their index pregnancy experienced an very short IPI compared to 4.1% of women who did not experience this RC behavior (*p* = 0.02).

**TABLE 2:** Weighted prevalence of short interpregnancy interval (< 12 months) by exposure to reproductive coercion in the 12 months before index pregnancy.

	Total *N* = 1935, *N* (%)	% with IPI < 12 months (95% CI)	*p* value
**Composite indicators**			
Any RC			0.09
No	1400 (72.7)	3.8 (2.6–5.7)	
Yes	535 (27.3)	5.8 (3.8–8.7)	
RC Severity			0.22
None	1400 (72.7)	3.8 (2.6–5.7)	
Less severe	325 (16.1)	5.5 (3.2–9.3)	
More severe	210 (11.2)	6.2 (3.5–10.9)	
**Individual RC behaviors**			
Told you not to use family planning			0.54
No	1459 (75.8)	4.2 (2.8–6.2)	
Yes	476 (24.2)	4.9 (3.2–7.6)	
Said he would leave you if you didn't get pregnant			0.86
No	1797 (92.7)	4.3 (3.1–6.1)	
Yes	138 (7.3)	4.7 (2.0–10.6)	
Told you he would have a baby with someone else if you didn't get pregnant			0.19
No	1850 (95.4)	4.2 (3.0–6.0)	
Yes	85 (4.6)	7.4 (3.3–15.8)	
Took away your family planning or kept you from going to the clinic			0.02
No	1835 (93.9)	4.1 (2.9–5.7)	
Yes	100 (6.1)	9.2 (4.7–17.3)	
Hurt you physically because you did not agree to get pregnant			0.04
No	1911 (98.4)	4.2 (3.0–5.9)	
Yes	24 (1.6)	14.3 (4.3–37.9)	

### 3.4 Cumulative probability of very short IPI

**FIGURE 1:**
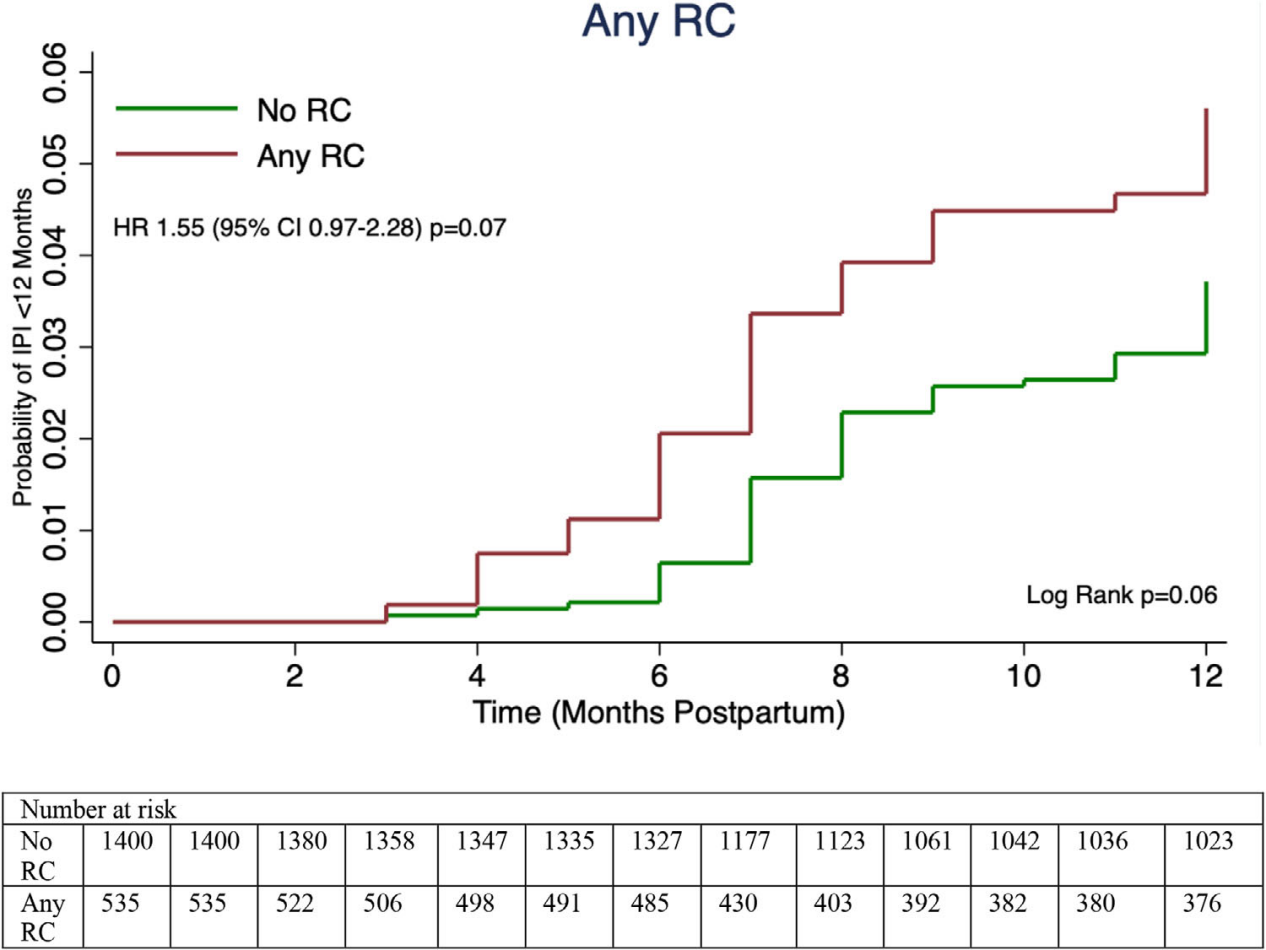
Cumulative probability of repeat pregnancy within 12 months of live birth, by experience of any reproductive coercion in the 12 months before index pregnancy.

Women who experienced any RC had marginally higher cumulative probabilities of very short IPI over the first 12 months postpartum compared to women who did not experience RC (Figure 1; *p* = 0.06). The cumulative probability of very short IPI by 12 months postpartum was marginally greater among women who experienced any RC (5.8%) compared to women who did not experience RC (3.8%), with a *p*‐value of 0.06, indicating a trend toward increased likelihood of very short IPI among women who experienced RC.

**FIGURE 2:**
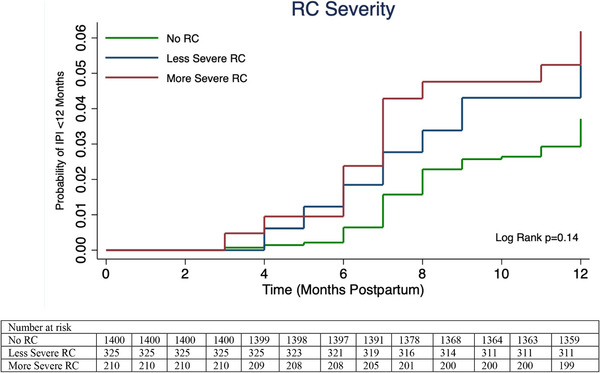
Cumulative probability of repeat pregnancy within 12 months of live birth, by severity of reproductive coercion in the 12 months before index pregnancy.

The cumulative probability of very short IPI increased with RC severity and trended toward significance; women who experienced more severe RC had higher probabilities of very short IPI over the first 12 months postpartum compared to women who did not experience RC (*p* = 0.14; Figure 2). At three months postpartum, the probability of short IPI was low across all groups, but slightly higher for women who experienced more severe RC compared to those with less severe RC or no RC. Over the course of the year, the probability of a subsequent pregnancy continued to increase, with the most substantial rise seen in the group with more severe RC. At 12 months, the failure probability was 6.2% for women with more severe RC, compared to 5.5% for those with less severe RC and 3.8% for women with no RC.

### 3.5 Hazard of very short IPI by exposure to RC

Table 3 presents the crude hazard ratios (HR) for the risk of very short IPI based on exposure to RC, adjusted for gestational age. In crude models, any RC was associated with a marginally higher risk of short IPI compared to no RC (HR: 1.57, 95% CI: 0.99–2.48, *p* = 0.06). When disaggregated by RC severity, there was a small, but non‐significant increase in risk of very short IPI among women who experienced less severe RC relative to those who did not experience RC (HR: 1.50, 95% CI: 0.93–2.99, *p* = 0.17). More severe RC marginally increased the hazard of very short IPI compared to no RC (HR: 1.66, 95% CI: 0.93–2.99, *p* = 0.09).

**TABLE 3:** Crude hazard of interpregnancy interval ≤12 months according to exposure to reproductive coercion, adjusted for gestational age at baseline (*N* = 1935).

	aHR	95% CI	*p*
Any RC (ref: none)			
Yes	1.57	(0.99–2.48)	0.06
RC Severity (ref: none)			
Less severe	1.50	(0.93–2.99)	0.17
More severe	1.66	(0.93–2.99)	0.09

*Note*: Crude models adjusted for gestational age at baseline to account for varying time at risk of RC.

When further adjusting for age, parity, religion, and fertility plans (Table 4), the hazard of very short IPI slightly attenuated but was still marginally higher among women who experienced any RC relative to women who did not experience RC (HR: 1.55, 95% CI: 0.97–2.48; *p* = 0.07). When disaggregating by RC severity, there was again a small, non‐significant increase in risk among women who experienced less severe RC (HR: 1.44, 95% CI: 0.80–2.59; *p* = 0.23), and a marginally higher risk of short IPI among women who experienced more severe RC behaviors (HR: 1.72, 95% CI 0.97–3.04, *p* = 0.06) compared to women who did not experience RC.

**TABLE 4:** Adjusted hazard of interpregnancy interval < 12 months according to exposure to reproductive coercion (*N* = 1935).

	Model 1 Binary RC				Model 2 Categorical RC		
	aHR	(95% CI)	*p*		aHR	(95% CI)	*p*
Any RC (ref: none)				RC Severity (ref: None)			
Yes	**1**.**55**	**(0**.**97–2**.**48)**	**0**.**07**	Less severe	1.44	(0.80–2.59)	0.23
				More severe	**1.72**	**(0.97–3.04)**	**0.06**
Age (ref: 25–29)							
15–24 years	**0**.**56**	**(0**.**30–1**.**05)**	**0**.**07**		0.56	(0.30–1.04)	0.07
30–49 years	0.79	(0.46–1.34)	0.38		0.78	(0.46–1.34)	0.37
Parity (ref: multiparous)							
Primiparous	1.62	(0.87–3.01)	0.12		1.64	(0.89–3.01)	0.11
Religion (ref: Orthodox)							
Muslim	1.52	(0.83–2.79)	0.17		1.51	(0.57–2.51)	0.63
Protestant/Other [1]	1.21	(0.57–2.55)	0.42		1.20	(0.57–2.51)	0.18
Index pregnancy intention (ref: intended)							
Unintended	0.82	(0.47–1.42)	0.47		0.82	(0.47–1.34)	0.47
Prospective fertility plans reported during index pregnancy (ref: wants more children)							
Wants no more children /don't know	0.86	(0.46–1.61)	0.63		0.85	(0.45–1.61)	0.63
Gestational age at enrollment	0.98	(0.88–1.10)	0.77		0.98	(0.88–1.10)	0.76

*Note*: Potential confounders included based on theoretical relevance and *p* < 0.20 in bivariate analyses (not shown). Models were weighted to account for the differential probability of selection and loss‐to‐follow‐up.

## 4 DISCUSSION

Numerous studies have examined determinants of short IPI in sub‐Saharan Africa [18, 24, 25, 26, 54], but to our knowledge, this is the first study to explore the relationship between partner‐perpetrated RC and short IPI. Our findings suggest an increased risk of subsequent very short IPI among women who experience RC before pregnancy. While any RC appears to increase the likelihood of very short IPIs, behaviors with explicit coercive intent—such as threats or interference with contraception (more severe RC)—may be most strongly associated with very short IPIs. Results suggest a potential dose‐dependent relationship between RC severity and short IPI which warrants further investigation.

Disaggregating RC by behavior severity to capture varying degrees of coercive intent enables a more nuanced understanding of the relationship between RC and short IPI. Less severe behaviors, such as discouragement of contraception, may lead some women to forgo postpartum contraceptive use due to fear of partner disapproval, increasing the likelihood of having a short IPI. On the other hand, women experiencing less severe RC may retain more autonomy to use contraception covertly or negotiate contraceptive use compared to those facing more severe RC—such as explicit interference or threats. This aligns with prior research in Ethiopia showing that more severe RC, but not less severe RC, is associated with decreased odds of contraceptive use [31]. Similarly, another study found that the likelihood of covert use increases RC severity [50]. While the less vs. more severe classification provides a framework for assessing relationships between RC and short IPI, we recognize that coercive experiences can vary in severity and are not always easily categorized. Further research is needed to understand how different RC behaviors shape contraceptive decision‐making and pregnancy spacing in the postpartum period.

The increasing magnitude of effect sizes with RC severity, alongside borderline significant *p*‐values, indicates a trend that warrants further investigation. When stratifying by severity, the failure rate for women with less severe RC (5.5% at 12 months) was higher than that of those with no RC (3.8%) and the hazard ratio for less severe RC indicated a higher risk of very short IPI relative to women who did not experience RC. However, this result was not statistically significant, suggesting that while the effect size was in the expected direction, the association between less severe RC and short IPI may be weaker than that of more severe RC. One explanation is that more severe RC behaviors have immediate, tangible consequences that disrupt a woman's ability to prevent pregnancy. In contrast, verbal discouragement may not immediately disrupt contraceptive use, as women may still be able to access and use contraception covertly, or the threat may not seem as immediate or urgent. Moreover, the dose‐dependent association between RC and short IPI suggests that the lack of statistical significance at the conventional 5% level may be due to lack of power rather than the absence of a true association. Taken together, study findings support further analysis in a larger cohort and for longer follow‐up.

Given the timeframe of RC in the present study, we suggest two hypotheses regarding potential mechanisms for the relationship between more severe RC experienced before the index pregnancy and subsequent very short IPI. First, severe RC is likely a marker for broader, ongoing power imbalances within a relationship [29] and controlling behavior may continue beyond the first reported instance [34, 35, 36, 55]. Therefore, RC before the index pregnancy may be a proxy measure for continued RC in the postpartum period. This is aligned with qualitative findings from Kenya, demonstrating some women experience pregnancy coercion and subsequent instances of increasing RC after the resulting birth, driven by a partner's desire to have another child soon [35]. Additional research is needed to examine the continuity of RC behaviors from pre‐pregnancy to postpartum and assess how RC behaviors change during this transition. Second, RC experience before an initial pregnancy may instill lasting fear of conflict or marital discord [56] that extends to subsequent reproductive decisions in the postpartum period. In other words, a partner's known opposition to family planning and discordant fertility intentions, which have previously led to the use of abusive tactics, may lessen a woman's self‐efficacy in decisions regarding pregnancy spacing and be a barrier to the use of PPFP [31, 32]. Unsurprisingly, uptake of postpartum contraception was lower among women who experienced a very short IPI (26.9%) than women who did not have a very short IPI (44.5%) in descriptive analyses. Further research should explore how women's past experiences of RC shape future decision‐making and reproductive autonomy in the postpartum period. In addition, research with men who have perpetrated RC is needed to understand how their behavior and attitudes evolve after childbirth and their motivations for continued RC shortly after having a child. This knowledge can inform strategies to engage men in supporting women to make autonomous and informed choices about pregnancy spacing.

While this study fills an important gap in understanding the relationship between partner‐perpetrated RC and subsequent short IPI, our study should be interpreted in the context of several limitations. First, despite a substantial sample size (*n* = 1935), the low prevalence of very short IPI—consistent with previous estimates in Ethiopia [37, 38]—reduced statistical power, making it difficult to detect smaller, yet meaningful, associations between RC and very short IPI at the conventional alpha level of 5% and contributed to imprecise effect size estimates. However, focusing solely on *p*‐values may overlook the practical significance of these findings for informing intervention strategies and future research; the increasing magnitude of effect sizes with RC severity suggests a potential dose‐response relationship that warrants further investigation.

Second, the follow‐up period was limited to 12 months postpartum, restricting our ability to capture IPIs occurring between 13 and 24 months, when the majority of short IPIs in Ethiopia occur [2]. Further research with extended follow‐up periods is needed to capture the full spectrum of short IPIs and further elucidate the long‐term impact of RC on pregnancy spacing. Despite this limitation, focusing on very short IPIs remains of public health significance, given their heightened risks for adverse maternal and neonatal outcomes [12, 13] compared to IPIs >12 months and < 24 months.

Third, direct measures of RC during the postpartum period were not captured. Therefore, the present research may not capture the experiences of RC most proximal to very short IPI. Still, findings support the hypothesis that pre‐pregnancy RC reflects a broader pattern of control within relationships, rather than an isolated event, and thus, pre‐pregnancy RC may be a proxy for ongoing RC. Despite this limitation, our findings remain relevant for understanding the lasting impact of RC on postpartum pregnancy spacing; the clear temporal relationship between RC and very short IPI is a key strength of the study design. Future research should incorporate longitudinal assessments of RC across multiple time points to better capture the dynamics of RC before, during, and after pregnancy.

Fourth, we did not capture women's retrospective pregnancy intentions for pregnancies occurring within the very short IPI, making it unclear whether these pregnancies were unintended. However, pregnancy intention measures collected retrospectively are often subject to social desirability bias. As a result, even if collected, these data may not have accurately reflected women's true intentions at the time of conception. While some women may have desired to conceive again shortly after giving birth, prior research suggests that short IPIs are often unintended. In our study, 20% of women who later had a very short IPI expressed during the index pregnancy that they did not want more children or were unsure, suggesting that some of the short IPI pregnancies may have been unintended, and some IPIs were shorter than women desired. Respecting women's reproductive autonomy is essential, including acknowledging that some may make informed decisions to have closely spaced pregnancies.

Preventing very short IPIs and safeguarding women's reproductive autonomy requires a comprehensive approach to RC prevention and response. Addressing RC necessitates multi‐sector efforts to shift patriarchal social norms that sustain abusive behaviors and position men as the primary decision‐makers in relationships [29]. Community‐based strategies that engage men in small group sessions and community dialogues aimed to foster positive engagement in contraceptive decisions, promote respect for women's choices and desired contraceptive use, agreement to use a male condom, and participation in contraceptive counseling [57] may be promising strategies in Ethiopia. Additionally, PPFP programs must recognize that abusive partners may be barriers to women achieving their desired number and spacing of pregnancies. Interventions like ARCHES (*Addressing Reproductive Coercion in Healthcare Setting*s) [58, 59]—offer a model for the Ethiopian context for training providers—including health extension workers, antenatal providers, and family planning staff—with the knowledge and skills to educate women on RC and support women who desire female‐controlled contraceptive methods that can be used without partner detection. Early implementation of strategies to help women initiate their preferred postpartum contraceptive methods may help mitigate the impact of RC on very short IPIs.

RC undermines women's autonomy in contraceptive and pregnancy‐related decision‐making. Experiencing RC before pregnancy and a subsequent very short IPI may signal ongoing constraints on reproductive control, limiting women's ability to achieve their desired pregnancy spacing. Since the first year after childbirth is a critical window to prevent unintended and closely spaced pregnancies, programmatic efforts must account for the potential impacts of severe RC on pregnancy spacing and prioritize strategies that empower women to make reproductive decisions free from coercion or violence.

## CONFLICT OF INTEREST STATEMENT

The authors declare no conflicts of interest.

## ETHICS STATEMENT

Institutional Review Boards at Addis Ababa University (Ref: AAUMF 01‐008) and the Johns Hopkins Bloomberg School of Public Health (FWA00000287) approved study procedures.

## Data Availability

The data supporting the findings of this study are publicly available in the Johns Hopkins Research Data Repository at https://archive.data.jhu.edu/dataset.xhtml?persistentId=doi:10.34976/y424‐s176.https://www.pmadata.org/data/request‐access‐datasets]

## Footnotes

a Administrative boundaries changed shortly after study enrollment. The Sidama Region and South West Ethiopia Region split off from SNNP in 2020 and 2021, respectively. As of 2022, Ethiopia consisted of 11 regional states and two chartered cities. For simplicity, we use the regional states and city administrations as they were at the time the 2019–2021 PMA Ethiopia cohort was conducted. Additionally, study enrollment in Tigray took place prior to the onset of civil conflict in 2020.
